# Clinical prognostic scores for patients with thymic epithelial tumors

**DOI:** 10.1038/s41598-019-54906-4

**Published:** 2019-12-09

**Authors:** Cecilia Veraar, Stefan Janik, Jürgen Thanner, Clarence Veraar, Mohamed Mouhieddine, Ana-Iris Schiefer, Leonhard Müllauer, Martin Dworschak, Walter Klepetko, Hendrik Jan Ankersmit, Bernhard Moser

**Affiliations:** 1 Department of Anesthesiology, General Intensive Care and Pain Medicine, Division of Cardiothoracic and Vascular Anesthesia and Intensive Care Medicine, Medical University of Vienna, Vienna, Austria; 2 Department of Otorhinolaryngology, Head and Neck Surgery, Medical University of Vienna, Vienna, Austria; 3 Division of Thoracic Surgery, Department of Surgery, Medical University of Vienna, Vienna, Austria; 4 Clinical Institute of Pathology, Medical University of Vienna, Vienna, Austria; 5 Head FFG Project “APOSEC“, FOLAB Surgery, Medical University of Vienna, Vienna, Austria

**Keywords:** Prognostic markers, Tumour biomarkers

## Abstract

Several inflammation-based prognostic scores emerged in various types of cancer to predict clinical outcomes. So far, no accurate pre-treatment scoring systems exist for patients with thymic epithelial tumors (TETs), comprising thymomas and thymic carcinomas (TCs). Therefore, we sought to test the prognostic value of different clinical composite scores and their components, identify optimal cut-off values for TETs as well as combine predictive components to new suitable prognostic scores. One hundred eighty-four patients with TETs undergoing surgical tumor resection were analyzed. A significant advantage in Freedom-from-Recurrence and/or Cause-specific survival (CSS) was evident for patients with high Advanced-Lung- Cancer-Inflammation-Index, low CRP-Fibrinogen-Score (CFS), low Glasgow-Prognostic-Score (GPS), low high-sensitivity-modified GPS, low TET-adapted GPS (TET-aGPS) and low Systemic-Immune-Inflammation Index. On multivariable analysis high TET-aGPS (HR = 14.9;p = 0.001), incomplete resection status (HR = 13.5;p = 0.001) and TC (HR = 26.0;p = 0.001) were significant independent prognostic factors for worse CSS. The CFS had the highest coefficient of determination (R^2^ = 0.188) to predict tumor recurrence of all composite scores, comprising CRP (R^2^ = 0.141) and fibrinogen (R^2^ = 0.158), the best single factor predictors. Inflammation-based prognostic scores and selected components are suitable to predict survival and/or tumor recurrence in TET patients undergoing primary surgery. Due to excellent long-term survival and frequent tumor recurrence, cut-off values were tailored to increase prognostic power.

## Introduction

Thymic epithelial tumors (TETs), including thymomas, thymic carcinomas (TCs), and thymic neuroendocrine tumors, are rare intrathoracic malignancies that occur in the prevascular mediastinum^1^. According to data from the U.S. cancer registry, the incidence of thymomas is 0.13 per 100,000 person-years^2^. TETs can be classified according to their histopathologic features or based on their level of invasiveness (T-classification), lymph node involvement (N-classification), and/or distant metastases (M-classification).

The Masaoka staging system, published in 1981 and modified by Koga *et al*. 1994, differentiates tumors according to their level of invasiveness from I to IV^3^. The World Health Organization (WHO) classification system, first published in 1999 and updated 2015, classifies TETs in type A, AB, B1, B2 and B3 thymomas, other rare subtypes (e.g. micronodular tymomas) and TCs on the basis of their histological morphology^4^. The proposed IASLC/ITMIG staging system first published in 2014 and adapted in 2016 is a new evidence-based TNM-staging system for thymic malignancies^5^.

The Masaoka-Koga staging system, the WHO histological classification and the recently proposed TNM staging system can only give reliable prognostic information according to pathological and histological features after surgical tumor resection. For patients with TETs no clinical scoring systems were implemented to predict clinical outcomes and identify patients at high risk of tumor recurrence or death before surgery and/or multimodal therapy.

In the last decades evidence was obtained that inflammation plays an essential role in carcinogenesis and tumor progression^6^. Several inflammation-based prognostic systems were proposed to predict clinical outcome in patients with cancer. Scoring systems such as the Advanced-Lung-Cancer-Inflammation-Index (ALI), the Glasgow-Prognostic-Score (GPS), the modified GPS (mGPS), the high-sensitivity mGPS (HS-mGPS) and the Systemic Immune-Inflammation Index (SII) emerged in many types of cancer but were never tested for TETs^7–10^. ALI was developed to assess systemic Inflammation and cancer cachexia based on patients’ body mass index (BMI), serum albumin concentration and neutrophil-to-lymphocyte ratio (NLR) from the time of diagnosis in patients with metastatic non-small-cell lung cancer (NSCLC) to detect cancer progression^8^. The GPS, mGPS and HS-mGPS are composed of two serum indicators: CRP as a marker for inflammatory responses and albumin reflecting nutritional status^9,11,12^. The scores are well explored for various clinical scenarios and types of tumors^13^. However, the mGPS and the HS-mGPS were established as more sensitive prognostic markers^14^. The HS-mGPS has shown prognostic superiority compared to the mGPS in patients with resectable gastric cancer^12^. The SII, first reported by Hu *et al*., integrates peripheral lymphocyte, neutrophil, and platelet counts to verify the inflammatory status and the immune response in patients with hepatocellular carcinoma. The SII was tested for colorectal cancer and gastric cancer^10,15^.

The American Society of Anesthesiologist’s (ASA) classification has established the most widely used patient risk assessment scheme in Anesthesiology developed in 1942 by Saklad to offer a simple categorization of a patient’s physiological status that can be helpful in predicting operative risk. In 1963 the ASA adopted the five-category system and an additional sixth category was later added^16^.

In our previous work we showed an association of systemic inflammatory proteins, such as CRP and fibrinogen and indices formed from inflammatory cell counts, such as NLR, with higher tumor stages and worse prognosis in patients with TETs^17,18^.

The present study was conducted in order to evaluate whether inflammation-based prognostic scores, proposed for cancers other than TETs, the components of these scoring systems alone, the widely adapted ASA score and our newly developed scoring systems, the CRP-fibrinogen score (CFS) and the TET- adapted GPS (using TET specific cut-off values to calculate the GPS) are suitable predictors of long-term outcome for patients with TETs undergoing surgery alone or in conjunction with multimodal therapy. Existing and/or tailored cut-off values were used to increase the prognostic power of the scores.

## Results

### Patient cohort

Demographic data of 184 evaluable patients was listed in Table 1. Forty-two percent of patients with thymomas and 84% of patients with TCs received multimodal treatment (i.e. regimens combining surgery with radiotherapy and/or chemotherapy). In particular, 12% and 37% of patients with thymomas compared to 56% and 49% of patients with TCs underwent neo-adjuvant and adjuvant therapy, respectively. Radical tumor resection with free resection margins (R0) could be achieved in 100% of all patients diagnosed with paraneoplastic Myasthenia Gravis (MG) and in 85% of patients without MG. Data to complete the ASA, ALI, CFS, GPS, HS-mGPS, TET-aGPS and SII score were available in 146, 114, 150, 149, 148, 149, and 139 patients, respectively.

**Table 1:** Basic demographic data, disease specific characteristics and treatment modalities.

**age** (yrs) mean *(median)* ± SD	56 (56) ± 15
**gender**	
female:male ratio *n (%)*	98:86 *(*5*3.3):(*4*6.7)*
**BMI** mean *(median)* ± SD	27 *(26)* ± *5*
**Histology**	
Thymoma *n (%)*	139 *(76)*
TC n *(%*)	45 (*25)*
**WHO Classification**	
A *n (%)*	20 (*11)*
AB *n (%)*	25 *(14)*
B1 *n (%)*	17 *(9)*
B2 *n (%)*	38 (*21)*
B3 *n (%)*	29 *(16)*
TC *n (%)*	45 *(24)*
MNT *n (%)*	7 *(4)*
TNET *n (%)*	3 *(2)*
**Residual tumor classification**	
R0 *n (%)*	164 *(89)*
R1 + 2 *n (%)*	20 (*11)*
**Myasthenia gravis**	
positive *n (%)*	48 (*26)*
negative *n (%)*	136 *(74)*
Tumor Stage*	
I-II *n (%)*	122 (*66)*
III-IV *n (%)*	62 *(34)*
**Multimodality-treatment**	
yes *n (%)*	97 (*53)*
no *n (%)*	87 (*47)*
**Neoadjuvant-therapy**	
yes *n (%)*	42 (*23)*
no *n (%)*	142 *(77)*
**Adjuvant-therapy**	
yes *n (%)*	73 *(60)*
no *n (%)*	111 *(40)*

*n* number of patients, *SD* standard deviation, *TC* thymic carcinoma, TNET thymic neuroendocrine tumor, *MNT* micronodular thymoma, Masaoka-Koga tumor stage, BMI Body mass index. *Masaoka-Koga tumor stage

### Scoring systems and MG

In our cohort, 48 patients with TETs were diagnosed with paraneoplastic MG and 136 patients were MG negative. The CFS revealed a statistically significant difference between both groups (p = 0.005). The majority of all patients with paraneoplastic MG (94.7%) were scored with CFS 0, whereas only 73% of all patients without MG had CFS 0. The ASA (p = 0.565), ALI (p = 0.141), GPS (p = 0.649), the HS-mGPS (p = 0.123), the TET-aGPS (p = 0.079) and the SII (p = 0.839) revealed no statistically significant difference among groups.

### Prognostic analysis: survival and recurrence

The median follow-up was 100 months. The 1-, 5- and 10-year overall survival (OS) rate of the entire cohort was 97%, 89%, and 82%, respectively.

The presence of TC, incomplete tumor resection (R1 + 2) and advanced tumor stages (III-IV) were associated with significantly worse OS (p = 0.001; p = 0.007; p = 0.032), CSS (p = 0.018; p = 0.017; p = 0.001), and FFR (p = 0.001; p = 0.001; p = 0.001), respectively.

**Figure 1:**
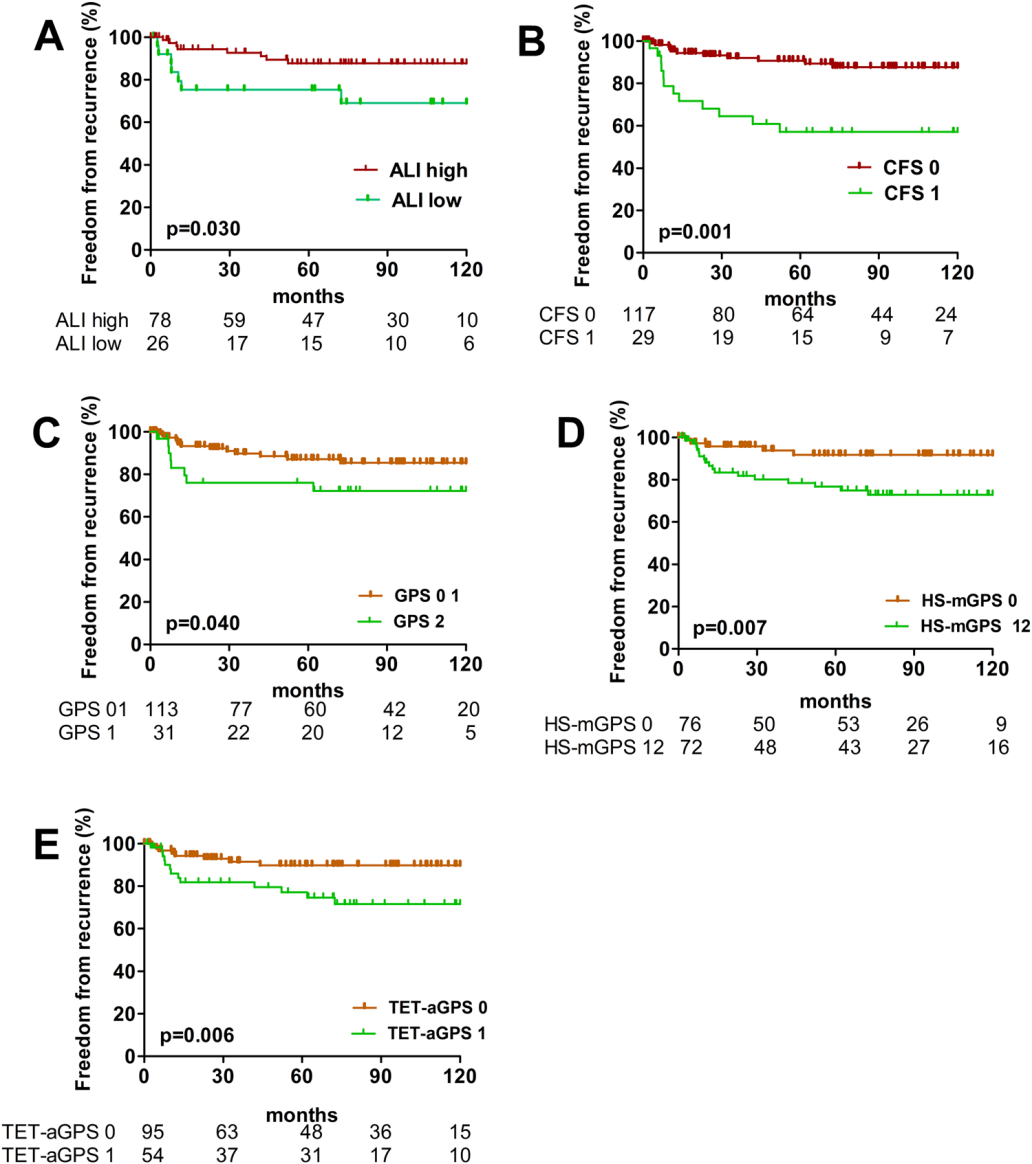
Freedom From Recurrence of composite scores. Freedom From Recurrence for patients divided into ALI low and ALI high, CFS 0 and 1, GPS 01 and 2, HS-mGPS 0 and 12, TET-aGPS 0 and 1 are shown in (**A–E**). *ALI* advanced lung cancer inflammation index, *CFS* CRP/Fibrinogen Prognostic score, *GPS* Glasgow Prognostic Score, *HS-mGPS* the high-sensitivity modified Glasgow Prognostic Score, *TET-aGPS* thymic epithelial tumor adapted Glasgow Prognostic Score.

**Figure 2:**
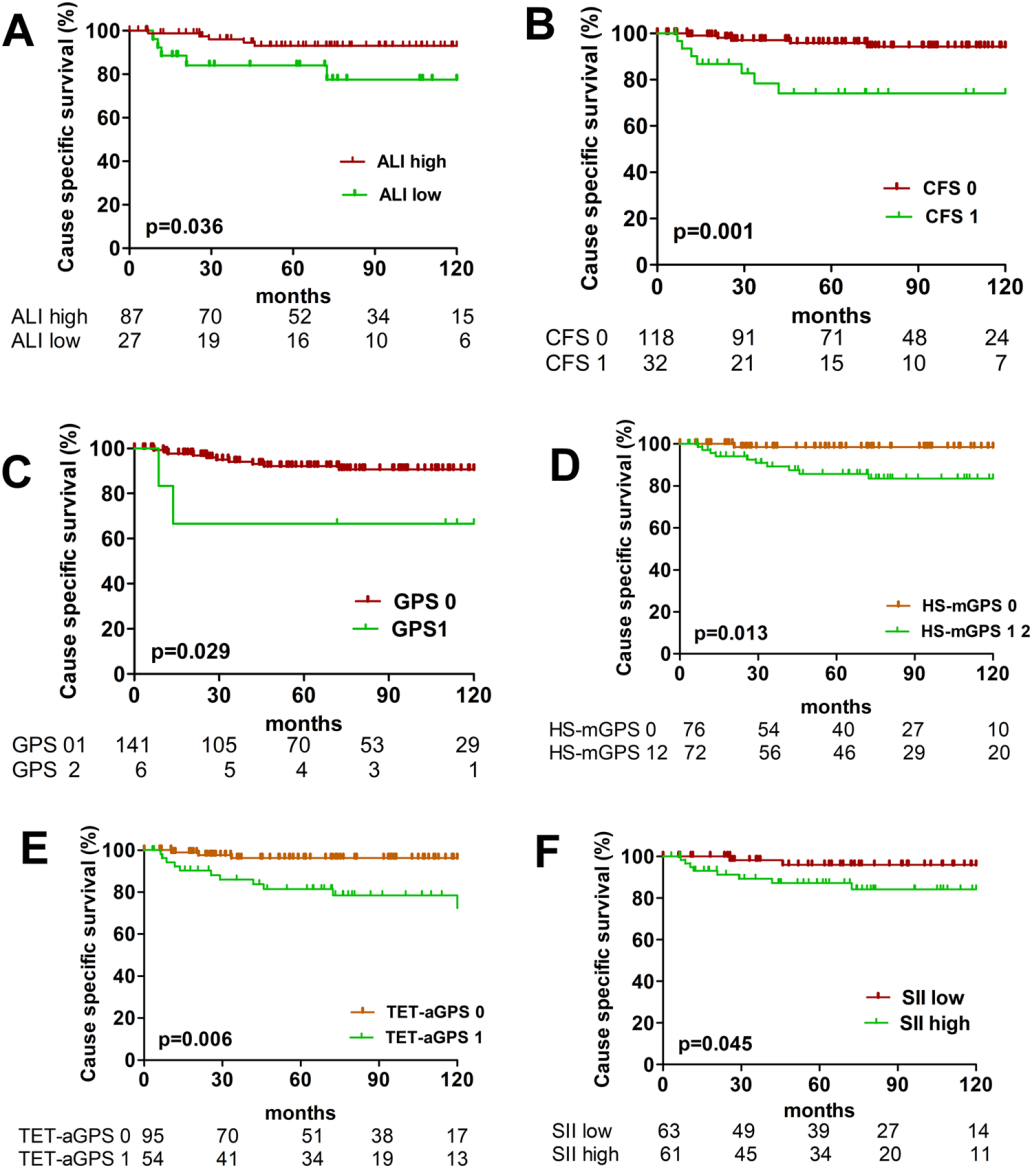
Cause specific survival of composite scores. Cause specific survival for patients divided into ALI low and ALI high, CFS 0 and 1, GPS 1 and 2, HS-mGPS 0 and 12, TET-aGPS 0 and 1 and SII low and high are shown in (**A-F**). *ALI* advanced lung cancer inflammation index, *CFS* CRP/Fibrinogen Prognostic score, *GPS* Glasgow Prognostic Score, *HS-mGPS* the high-sensitivity modified Glasgow Prognostic Score, *TET-aGPS* thymic epithelial tumor adapted Glasgow Prognostic Score*, SII* systemic immune-inflammation index.

Additionally, we assessed the ability of each scoring system to predict clinical outcome. There were no significant differences between patients allocated into ASA 12 and ASA 34 score in OS (p = 0.348), CSS (p = 0.240) and FFR (p = 0.849), respectively. Conversely, patients with high ALI (>26.1) had significantly better CSS (p = 0.036) and FFR (p = 0.030), while OS was not significantly affected (p = 0.229). Between patients classified into CFS 0 and CFS 1 subgroups, there was a significant difference in CSS (p < 0.001) and FFR (p < 0.001), respectively. The GPS was statistically significant in predicting FFR (p = 0.009), patients with HS-mGPS 0 had statistically significant better CSS (p = 0.013) and FFR (p = 0.002) compared to patients with HS-mGPS 1 + 2, TET-aGPS 0 was associated with better OS (p = 0.034), CSS (p = 0.006) and FFR (p = 0.006) compared to patients with TET-aGPS 1. The SII revealed only a significant difference in predicting CSS (p = 0.045), respectively (Table 2, Figs. 1 and 2).

**Table 2:** Outcome analysis: survival and recurrence.

	Overall survival			Cause specific survival			Freedom From Recurrence		
	5 year	10 year	*p*	5 year	10 year	*p*	5 year	10 year	*p*
**Postoperative predictors**									
**Histology**									
Thymoma	93.6	86.3	**0.001**	98.6	97.1	**0.018**	91.7	89.3	**0.001**
TC	73.3	66.7		77.8	75.6		65.9	61.4	
**Residual tumor classification**									
R0	91.0	83.5	**0.007**	94.4	93.3	**0.017**	87.8	85.2	**0.001**
R1 + 2	78.9	65.0		84.2	80.0		62.5	56.3	
Tumor Stage*									
I-II	93.3	86.1	**0.032**	96.7	96.7	**0.001**	93.0	92.1	**0.001**
III-IV	80.4	72.6		85.7	82.3		69.6	62.5	
**Preoperative predictors**									
**ASA**									
1	100	100	**0.348**	100	100	**0.240**	88.9	88.9	0.849
2	85.9	81.3		93.8	90.6		88.5	85.2	
3	89.4	80.3		93.9	93.9		88.5	86.9	
*4*	71.4	57.1		71.4	71.4		71.4	71.4	
**ALI**									
high	87.4	80.5	**0.229**	94.3	94.3	**0.036**	90.0	89.9	**0.030**
low	81.5	70.4		85.2	81.5		76.9	73.1	
**CFS**									
low	91.5	83.1	**0.074**	96.6	95.8	**0.001**	92.0	90.2	**0.001**
high	71.9	71.9		78.1	78.1		58.6	58.6	
**GPS**									
0	88.7	88.9	**0.776**	93.9	93.0	**0.074**	88.9	88.0	**0.009**
1	84.6	80.9		92.3	92.3		84.0	80.0	
2	66.7	66.7		66.7	66.7		50.0	40.0	
**HS-mGPS**									
0	90.8	88.2	**0.172**	97.4	97.4	**0.013**	93.1	93.1	**0.002**
1	84.8	72.7		89.4	87.9		80.6	77.4	
2	66.7	66.7		66.7	66.7		50.0	40.0	
**TET-aGPS**									
0	91.6	86.3	**0.034**	96.8	96.8	**0.006**	91.1	91.1	**0.006**
1	79.6	70.4		85.2	83.3		76.5	72.0	
**SII**									
low	88.9	79.4	**0.690**	96.8	96.8	**0.045**	91.2	91.2	0.056
high	85.2	78.7		88.5	86.9		81.4	79.3	

Three different parameters were tested in the Kaplan Meier survival analysis: overall survival, cause specific survival and freedom from recurrence. Accepted clinical predictors such as histology, tumor stage and residual tumor classification were tested along with ASA and composite clinical scores. Cut-offs: The Youden Index was employed to define the optimal ALI cutoff of 26.1 and 655 for the SII; the median pretreatment CRP value of 3 mg/L was used to dichotomize patients into high and low CRP groups for the CFS score.

*ASA* American Society of Anesthesiology classification of Physical Health, *ALI* advanced lung cancer inflammation index, *CFS* CRP/Fibrinogen Prognostic score, *GPS* Glasgow Prognostic Score, *HS-mGPS* the high-sensitivity modified Glasgow Prognostic Score, *TET-aGPS* thymic epithelial tumor adapted Glasgow Prognostic Score, *SII* systemic immune-inflammation index, *Masaokoa- Koga tumor stage.

In addition, we performed separate analyses of thymoma and TC patients for each of the scoring systems to predict clinical outcomes. When thymoma patients were analyzed separately, only the GPS and the TET-aGPS displayed statistically significant differences in FFR among groups. GPS 0 was associated with significantly better FFR (p = 0.001) compared to patients scored with GPS 1 + 2, however there was no difference for CSS (p = 0.823), respectively. TET-aGPS 0 was associated with significantly better CSS (p = 0.050), but not FFR (p = 0.075) compared to patients with TET-aGPS 1.

There were no significant differences between thymoma patients allocated into ASA 12 and ASA 34 score in CSS (p = 0.073) and FFR (p = 0.092), in high ALI (>26.1) in CSS (p = 0.551) and FFR (p = 0.399), in patients classified into CFS 0 in CSS (p = 0.109) and FFR (p = 0.229) compared to CFS 1, in patients with HS-mGPS 0 compared to HS-mGPS1 + 2 in CSS (p = 0.148) and FFR (p = 0.301) and in SII in predicting CSS (p = 0.887) and FFR (p = 0.683), respectively.

In the separate analyses of TCs, no statistically significant differences regarding the investigated scores could be demonstrated. There were no significant differences between ASA 12 and ASA 34 patients in CSS (p = 0.273) and FFR (p = 0.243), in high ALI (>26.1) in CSS (p = 0.681) and FFR (p = 0.682), in CFS 0 and CFS 1 subgroups, in CSS (p = 0.250) and FFR (p = 0.114), in GPS in predicting CSS (p = 0.253) and FFR (p = 0.895), in HS-mGPS 0 compared to HS-mGPS1 + 2 patients in CSS (p = 0.215) and FFR (p = 0.122), in TET-aGPS 0 compared to patients with TET-aGPS 1 in CSS (p = 0.104) and FFR (p = 0.202) and in SII in predicting CSS (p = 0.197) and FFR (p = 0.374), respectively.

### Univariable and multivariable analysis of the scoring systems

Univariable analysis revealed significantly worse CSS in patients with TC (HR = 11.1; p < 0.001), high CFS (HR = 5.94; p = 0.002), high Masaoka-Koga tumor stage (HR = 5.52; p = 0.003), high GPS (HR = 4.90; p = 0.040), high HS-mGPS (HR = 4.87; p = 0.041), high TET-aGPS (HR = 5.18; p = 0.014) and low ALI (HR = 3.47; p = 0.049), respectively. The ASA and the SII revealed no statistically significant difference among the tested subgroups (Table 3).

**Table 3:** Univariable and multivariable Cox regression analysis of basic demographics, clinical composite scores and pathophysiological data.

	Univariable Model				Multivariable Model			
	HR	*p*	Lower	Upper	HR	*p*	95% CI	
**Cause Specific Survival**								
Sex (Male)	3.65	**0.026**	1.16	11.4				
Age years (<56 vs. >56)	1.55	0.398	0.56	4.28				
Histology (TC vs. Thymoma)	11.1	**0.001**	3.43	34.4	26.0	**0.001**	4.50	151.3
Resection Status (R1–2 vs. R0)	3.70	**0.025**	1.17	11.7	13.5	**0.018**	1.56	117.4
Myasthenia Gravis (No)	5.65	0.094	0.74	43.0				
Tumor Stage^*^ (III–IV vs. I + II)	5.52	**0.003**	1.75	17.3	2.21	0.407	0.33	14.59
ASA (III–IV vs. I + II)	0.98	0.983	0.31	3.06				
ALI (low vs. high)	3.47	**0.049**	1.00	12.0	2.43	0.321	0.42	14.08
CFS (1 vs. 0)	5.94	**0.002**	1.88	18.7	1.60	0.539	0.356	7.22
GPS (2 vs. 0 + 1)	4.90	**0.040**	1.07	22.3				
TET-aGPS (1 vs. 0)	5.18	**0.014**	1.40	19.1	14.9	**0.017**	1.63	137.4
HS-mGPS (2 + 1 vs. 0)	4.87	**0.041**	1.06	22.2				
SII (high vs. low)	4.28	0.066	0.01	20.1				
**Freedom From Recurrence**								
Sex (Male)	2.37	**0.022**	1.13	4.94				
Age years (<50 vs. >50)	1.92	0.070	0.94	3.88				
Histology (TC vs. Thymoma)	4.45	**0.001**	2.19	9.04	28.2	**0.001**	6.22	128.1
Resection Status (R1–2 vs. R0)	3.55	**0.003**	1.53	8.26	13.9	**0.003**	2.52	77.29
Myasthenia Gravis (No)	3.77	**0.029**	1.14	12.4				
Tumor Stage^*^ (III–IV vs. I + II)	4.86	**0.001**	2.37	10.5	4.78	**0.042**	1.06	21.59
ASA (III–IV vs. I + II)	1.05	0.906	0.43	2.54				
ALI (low vs. high)	2.92	**0.038**	1.06	8.07	2.89	0.119	0.76	11.04
CFS (1 vs. 0)	4.56	**0.001**	2.01	10.3	1.18	0.850	0.30	4.31
GPS (2 vs. 0 + 1)	5.13	**0.009**	1.50	17.5				
TET-aGPS (2 + 1 vs. 0)	3.17	**0.009**	1.33	7.57	3.44	0.093	0.81	14.57
HS-mGPS (1 vs. 0)	3.58	**0.012**	1.32	9.73				
SII (high vs. low)	2.65	0.066	0.93	7.55				

Multivariable cox regression for CSS and FFR was performed with all significant parameters (p < 0.05) of the univariable analysis for CSS, despite of sex. Only the most significant GPS score of the univariable analysis for CSS was included in the multivariable analysis of CSS and FFR.

*ASA* American Society of Anesthesiology classification of Physical Health, *ALI* advanced lung cancer inflammation index, *CFS* CRP/Fibrinogen Prognostic score, *GPS* Glasgow Prognostic Score, *HS-mGPS* the high-sensitivity modified Glasgow Prognostic Score, *TET-aGPS* thymic epithelial tumor adapted Glasgow Prognostic Score, *SII* systemic immune-inflammation index, *w/o* without, ^*^Masaoka- Koga tumor stage.

The presence of TC, resection status R1 + R2 and patients with high TET-aGPS remained statistically significant predictors of worse CSS (HR = 26.0; p < 0.001), (HR = 13.5; p = 0.018) and (HR = 14.9; p = 0.017) in multivariable analyses.

Regarding FFR, univariable analyses showed that the risk of recurrence was significantly increased in men (HR = 2.37; p = 0.02), in patients with TC (HR = 4.45; p < 0.001), resection status R1 + R2 (HR = 3.55; p = 0.029), in patients with absence of MG (HR = 3.77; p = 0.029), in patients with Masaoka-Koga stage III-IV (HR = 4.86; p < 0.001), low ALI (HR = 2.92; p = 0.038), high CFS (HR = 4.56; p = 0.001), high GPS (HR = 5.13; p = 0.009) and high TET-aGPS (HR = 3.17; p = 0.009). Multivariable analyses remained statistically significant for patients with TC (HR = 28.24; p < 0.001), for patients with incomplete resection status R1 + R2 (HR = 13.96; p = 0.003) and for patients with Masaoka-Koga stage III-IV (HR = 4.78; 0.042), while none of the clinical composite scores represented an independent predictive factor for FFR (Table 3).

Univariable analysis for thymoma revealed significantly worse CSS in patients with resection status R1 + R2 (HR = 14; p = 0.009) and high Masaoka-Koga tumor stage (HR = 9.7; p = 0.049), respectively. Clinical prognostic scores, sex, age and the presence of Myasthenia Gravis did not affect CSS. Regarding FFR, univariable analyses showed that the risk of recurrence was significantly increased in men (HR = 3.01; p = 0.048), resection status R1 + R2 (HR = 4.57; p = 0.021), in patients with Masaoka-Koga stage III-IV (HR = 6.68; p = 0.001), and high GPS (HR = 85.9; p = 0.002). Univariable analysis for TCs did not reveal significant differences in CSS and FFR”.

### Univariable analysis of the components of the scoring systems

To assess the strength of all individual components of the scoring systems, univariable analyses were performed.

Albumin, NLR and BMI were components of the ALI. Patients with albumin (<35 g/L) had a higher risk of recurrence (HR = 3.91; p = 0.028), while CSS was not affected. Additionally, patients with high NLR (>3.4) had a significantly worse CSS (HR = 3.62; p = 0.046) and FFR (HR = 2.59; p = 0.043). BMI did neither influence CSS nor FFR statistically significant.

CRP and fibrinogen were the components of the CFS. Patients with CRP (>0.3 mg/dL) showed significantly worse CSS (HR 4.9; p = 0.042) as well as a worse FFR (HR 3.5; p = 0.013). Similarly, patients with high fibrinogen (>452 mg/dL) showed significantly worse CSS (HR 5.7; p = 0.003) and FFR (HR 4.4; p = 0.001).

The GPS comprises CRP (>1 mg/dL) and albumin (<35 g/L) serum levels. CRP > 1 mg/dl did neither impact the CSS nor the FFR. The HS-mGPS was composed of previously described variables albumin (<35 g/L) and CRP (>0.3 mg/L). The TET-aGPS, however, uses different threshold levels for albumin (<44.4 g/L) and CRP (>0.3 mg/L). Patients with albumin (<44.4 g/L) had significantly worse FFR (HR = 4.38; p = 0.001).

The SII is composed of absolute lymphocyte, platelet and neutrophils counts. A higher count for lymphocytes (>4.2) was associated with worse CSS (HR = 9.75; p = 0.031) and FFR (HR = 4.36; p = 0.009). However, absolute platelet and neutrophil counts had no statistically significant impact on CSS and FFR (Table 4). Univariable analysis of the single factors did not reveal significant differences in CSS and FFR for thymoma and TCs.

**Table 4:** Univariable Cox regression analysis of the single components used in composite scores.

Univariable Model				
	HR	*p*	95% CI	
**Cause Specific Survival ALI**				
Albumin (<35 g/L)	4.12	0.067	0.90	18.50
BMI (<26 kg/m^2^)	1.16	0.782	0.40	3.34
NLR (>3.4)	3.62	**0.046**	1.02	12.84
**CFS**				
CRP (>0.3 mg/L)	4.85	**0.042**	1.06	22.14
Fibrinogen (>452 mg/dL)	5.73	**0.003**	1.82	18.14
**GPS**				
CRP (>1 mg/dL)	1.81	0.329	0.54	6.04
Albumin (<35 g/L)				
**HS-mGPS**				
Albumin (<35 g/L)				
CRP (>0.3 mg/L)				
**TET-aGPS**				
CRP (>0.3 mg/L)				
Albumin (<44.4 g/L)	3.00	0.156	0.65	13.71
**SII**				
Absolute neutrophil count (<4.2)	1.69	0.412	0.47	6.02
Platelet count (<266 G/L)	1.50	0.503	0.45	4.99
Absolute lymphocyte count (<1.9)	9.75	**0.031**	1.23	77.0
**Freedom From Recurrence ALI**				
Albumin (<35 g/L)	3.91	**0.028**	1.15	13.2
BMI (<26.1 kg/m^2^)	1.63	0.235	0.72	3.66
NLR (>3.4)	2.59	**0.043**	1.02	6.54
**CFS**				
CRP (>0.3 mg/dL)	3.53	**0.013**	1.30	9.57
Fibrinogen (>452 mg/dL)	4.38	**0.001**	1.04	5.42
**GPS**				
CRP (>1.0 mg/dL)	2.29	0.052	0.99	5.29
Albumin (<35 g/L)				
**HS-GPS**				
Albumin (<35 g/L)				
CRP (>0.3 mg/dL)				
**TET-aGPS**				
CRP (>0.3 mg/dL)				
Albumin <44.4 g/L)	4.38	**0.001**	1.04	5.42
**SII**				
Neutrophil count (<4.2)	1.30	0.559	0.53	3.21
Platelet count (<266 G/l)	1.43	0.411	0.60	3.38
Lymphocyte count (<1.9)	4.36	**0.009**	1.44	13.16

*ALI* advanced lung cancer inflammation index, *CFS* CRP/Fibrinogen Prognostic score, *GPS* Glasgow Prognostic Score, *HS-mGPS* the high-sensitivity modified Glasgow Prognostic Score, *TET-aGPS* thymic epithelial tumor adapted Glasgow Prognostic Score, *SII* systemic immune-inflammation index, 95*% CI 95*% Confidence interval, *NLR* neutrophil lymphocyte ratio, *BMI* body mass index, *Neutrophil count* Absolute neutrophil count, *Lymphocyte count* absolute Lymphocyte count.

### Scoring systems as prognostic markers

As shown in Table 5, we assessed the sensitivity, specificity, the positive and the negative predictive value (PPV and NPV) for all scoring systems for predicting tumor recurrence. The HS-mGPS had the highest sensitivity of 78% and the highest NPV of 93%. The GPS revealed the highest specificity of 99% and the highest PPV of 80%, respectively. Binary logistic regression analysis revealed that the CFS (R^2^ = 0.188; p = 0.001), the GPS (R^2^ = 0.127; p = 0.005), the HS-mGPS (R^2^ = 0.122; p = 0.003) and the TET-aGPS (R^2^ = 0.117; p = 0.020) were statistically significant for predicting tumor recurrence.

**Table 5:** Prognostic power of inflammation-based scoring systems and the single components in predicting tumor recurrence.

	R^2^	*p*	OR	Sensitivity (%)	Specificity (%)	PPV (%)	NPV (%)
**Composite clinical scores**							
ASA	0.000	0.944	1.03	50.0	50.8	14.7	85.7
ALI	0.054	0.063	2.86	46.7	78.9	26.9	89.9
CFS	0.188	**0.001**	7.46	54.2	86.3	41.4	90.2
GPS	0.127	**0.005**	25.5	18.2	**99.1**	**80.0**	86.5
HS-mGPS	0.122	**0.003**	4.92	**78.3**	57.8	26.9	**93.1**
TET-aGPS	0.117	**0.020**	4.39	65.2	70.1	30.0	91.1
SII	0.044	0.093	2.45	70.6	53.1	20.7	91.2
**Components of the clinical scores**							
Albumin (<35 g/L)	0.090	**0.006**	11.0	**66.7**	85.5	16.7	**98.3**
BMI (<26.1 kg/m^2^)	0.017	0.214	1.72	25.3	83.3	60.6	52.4
NLR (>3.4)	0.051	0.068	2.58	32.1	84.5	45.0	75.9
**CFS**							
CRP (>0.3 mg/dL)	0.141	**0.001**	5.60	28.6	**93.3**	**80.0**	58.3
Fibrinogen (>452 mg/dl)	0.158	**0.001**	6.19	41.9	89.6	52.0	85.1
**GPS**							
CRP (>1.0 mg/dL)	0.048	**0.038**	2.37	31.3	87.5	41.7	81.7
Albumin (<35 g/L)							
**HS-mGPS**							
CRP (>0.3 mg/dL)							
Albumin (<35 g/L)							
**TET-aGPS**							
CRP (>0.3 mg/dL)							
Albumin (<44.4 g/L)	0.011	0.336	1.59	18.9	87.3	70.8	39.7

*R*^2^ R square, *OR* odds ratio, *PPV* positive predictive value, *NPV* negative predictive value, *ASA* American Society of Anesthesiology classification of Physical Health*, ALI* advanced lung cancer inflammation index, *CFS* CRP/Fibrinogen Prognostic score, *GPS* Glasgow Prognostic Score, *HS-mGPS* the high-sensitivity modified Glasgow Prognostic Score, *TET-aGPS* thymic epithelial tumor adapted Glasgow Prognostic Score, *SII* systemic immune-inflammation index.

Further, the single components of the statistically significant scores of the binary logistic regression analysis were analyzed. Albumin (<35 g/L) (R^2^ = 0.090; p = 0.006), CRP (>0.3 mg/dL) (R^2^ = 0.141; p = 0.001), fibrinogen (R^2^ = 0.158; p = 0.001) and CRP (>1.0 mg/dl) (R^2^ = 0.048; p = 0.038) were statistically significant. The best single factor predictors were CRP (>0.3 mg/dL) and fibrinogen (>452 mg/dL), respectively (Table 5).

## Discussion

Assessment of patient prognosis is an important part of daily clinical practice. Gospodarowicz *et al*. divided prognostic factors into those that are tumor-related, host-related or environment-related^19^. In our study, newly developed host-related scoring systems based on the patients’ inflammatory status were compared with already well-established tumor-related prognostic factors for TETs, such as the histological WHO classification and the pathological Masaoka-Koga staging system. In our population we assume a negligible effect of environment-related prognostic factors such as choice, quality and access to treatment on the prognosis of TETs. Since the study was performed as a single center study each patient obtained the same quality of treatment. However, the choice of treatment was dependent on histological, pathological and clinical presentation.

The ability of the ASA to independently predict post-operative medical complications and mortality across a variety of surgical specialties and procedures has been reported for 2,297,629 cases^20^. In our study no significant difference in OS, CSS and FFR between participants with high or low ASA scores could be found. Our findings might be biased due to relatively young participants (median 55.9 ± 15) with only few comorbidities. The predictive power of our findings might be limited by the small sample size for the analysis of the ASA score. Only seven patients were scored with ASA 4 and only ten patients were classified with ASA 1. All patients in this study underwent thoracic surgery with curative intention. A selection bias might have occurred as patients with unresectable tumors or those with comorbidities incompatible to anesthesia and/or surgery were not part of this study. As a conclusion, the ASA score, which was designed to estimate perioperative risk, has no role in predicting long-term outcomes in patients with TETs.

The ALI with a cut-off of 18 was exclusively developed for patients with advanced stage IV NSCLC to assess ongoing systemic inflammation. Most of the patients already had metastatic disease at the time of diagnosis^21^. In general, NSCLC stage IV is a very aggressive malignancy with a 5-year OS of 13%^22,23^. ALI > 18, representing lower systemic inflammation was associated with better outcome in patients with NSCLC. In 2018 Tomita *et al*. identified the prognostic significance of ALI in patients with operable NSCLC. In this cohort the optimal cut-off value of ALI was defined as 37.6, and those patients allocated to the low-ALI group had significantly poorer survival rates^8^. In contrast to NSCLC, 5- and 10-year OS rates of all tumor stages of TETs vary between 85% and 73% for thymomas and between 61% and 37% for TCs, respectively^24,25^. In our study, patients of all tumor stages were included and 19% were assessed with advanced Masaoka-Koga stage IV. Therefore, the cut-off value was modified, using the Youden-Index. Accordingly, patients with high ALI scores (>26.1) had significantly better 10-year CSS and FFR. In our study, single factor analysis of the components of the ALI score had only shown statistically significant results for NLR to predict CSS and significant results for Albumin and NLR to predict FFR. Our results were in line with Sarraf *et al*. who described NLR as a good marker for systemic inflammation to predict poor outcome in patients with different types of malignancies^26^. An elevated NLR implies an increased neutrophil count and/or a decreased lymphocyte count as well as relative lymphopenia. Eerola *et al*. described the key role of lymphocytes in killing cancer cells and regulating the proliferation, apoptosis, angiogenesis and metastasis of cancer^27,28^. However, NLR had less predictive power than the ALI as a composite score regarding R^2^, sensitivity, specificity, NPV and PPV.

Albumin alone had a higher R^2^, sensitivity, specificity and NPV than the ALI, as a composite score out of albumin, BMI and NLR. Decreased serum albumin concentration due to reduced albumin synthesis caused by malnutrition and cancer induced inflammation has been described as an independent prognosticator of survival in various malignancies^29,30^. However, tumor related malnutrition is rarely seen in TETs. Only seven patients in our cohort had albumin values <35 g/L. All of them had advanced tumor stages, including five patients with TC and two patients with WHO stage B3 thymomas. BMI had no considerable prognostic relevance. Altogether, decreased serum albumin concentration provides prognostic information in advanced tumor stages in TETs. NLR and albumin are simple, routinely available predictors of survival and recurrence. Therefore, calculating the ALI score including BMI as a component without informative value for patients with TETs is not recommended.

Our previous studies demonstrated that pretreatment fibrinogen and CRP concentrations were associated with higher tumor stages and worse clinical outcome for TETs^17^. Moreover, we have shown that CRP and fibrinogen represented suitable markers to predict clinical outcome. The newly developed CFS combined preoperative fibrinogen und CRP concentrations to an easy practicable scoring system for everyday clinical practice. The CFS combines only predictive, protein-based parameters for the survival outcome of TETs. In contrast, the other already existing inflammation-based scoring systems contain irrelevant components for the prognosis of TETs such as the BMI.

The CFS had the highest coefficient of determination (R^2^ = 0.188) compared to all the other inflammatory prognostic systems. Further, the combination of both acute-phase proteins revealed a higher prognostic benefit compared to the prognostic power of CRP (R^2^ = 0.141) and fibrinogen (R^2^ = 0.158) alone.

Initially, GPS was applied to determine the prognosis of patients with inoperable lung cancer^22^. More than 60 studies (>30,000 patients) have validated the prognostic value of the GPS in patients with different types of tumors. The scoring system was identified as an excellent prognostic factor to discover systemic inflammation and malnutrition^31–34^. However, the GPS has never been tested for TETs. In our cohort the GPS had the highest sensitivity of 99% compared to all the other prognostic scores. As already discussed above, as the GPS score includes albumin along with CRP (>1 mg/dL), the score was especially useful to detect patients with TETs at high risk iof recurrence and death in advance. However, CRP (>1 mg/dL) as a cut-off for patients with TETs had not enough prognostic power (R^2^ = 0.048). In several studies, experts have already suggested that a lower threshold for CRP (cutoff value: 0.3 mg/dL) may enhance the prognostic value of the GPS in tumor patients. Therefore, the HS-mGPS has been proposed^35^. In our cohort the HS-mGPS was superior to the GPS in predicting OS, CSS and FFR, besides sensitivity and NPV were increased. As the median CRP (0.3 mg/dl) of our cohort was already validated as a good predictive marker, the TET-aGPS score was developed using the Youden Index to find the best cut-off value for albumin (<44.4 g/L). The TET-aGPS was the only significant independent prognostic factor in the multivariable analysis next to the postoperative predictors such as histology and resection status. Furthermore, in the univariable analysis the HR (4.38; p = 0.001) for albumin (<44.4 g/L) was higher compared to the HR (3.91;p = 0.028) of albumin (<35.5 g/L), respectively. Accordingly, the TET-aGPS is the GPS of choice to predict survival and recurrence for patients with TETs.

The SII first described in 2014, was constructed based on absolute lymphocyte, neutrophil, and platelet counts and was shown to be an independent predictor of postoperative recurrence and survival for patients with hepatocellular carcinoma (HCC)^15^. A cut-off value of 355 was proposed to dichtotomize patients into low and high groups. In our cohort 355 did not reveal a significant difference among low and high SII regarding CSS and FFR. The difference may be explained by the fact that patients with HCC may have more comorbidities than patients with TETs. Already more than 90% of all HCCs were found in a cirrhotically altered liver^36^. Therefore, liver function is an important determinant of survival. The severity of liver cirrhosis is prognostically more relevant than the presence of HCC. Further, OS appeared to have greater predictive power than CSS or FFR^37^. Herein, SII was calculated from serum blood parameter concentrations, determined before surgery. Our SII score cut-off > 655 was associated with significant worse 5- and 10-year CSS. We identified lymphocytes as an independent predictor of worse clinical outcome in the univariable analysis; while high platelet counts and decreased neutrophils did not correspond with worse clinical outcome. Conversely, several former studies have reported an association of increased platelet counts and a decrease in OS and poor prognosis of cancer^17,38^. Considering the absolute lymphocyte count alone is superior to the calculation of the SII in patients with TETs.

Several studies have shown that patients with TETs diagnosed with paraneoplastic MG have an equal or even better survival than patients without MG^39,40^. This result emerged out of improved management of MG, including earlier diagnosis of TETs due to their stricter follow-up^41^. These findings were in line with our results: patients with paraneoplastic MG had a significantly better OS and a significantly lower CFS. In our previous work we have already shown that patients with paraneoplastic MG had significantly lower fibrinogen serum concentrations, a component of the CFS^17,18^. However, CRP serum concentration (the second component of the CFS) did not differ between patients with or without MG. In our cohort, 68% of all females were diagnosed with MG, whereas only 31% of all men were MG positive. These findings might explain that men had significantly worse CSS and FFR in the univariable analysis.

In conclusion, we recommend analyzing the CFS, the TET-aGPS, absolute lymphocyte count and the NLR alone in preoperative routine work-up to better predict clinical survival and tumor recurrence in patients with TETs. The CFS and the TET-aGPS are both easy to calculate and therefore simple to implement in daily routine work. Calculating scoring systems developed from cancers other than TETs may integrate information without predictive value.

Because sample size is a major issue in a rare disease such as TETs, performing and interpreting the results of a multivariable analysis must be done with caution^42^. Due to insufficient sample size, small or medium-sized effects cannot be detected. In separate analyses of thymoma and TC patients, sample size was further reduced. The TETa-GPS was the only clinical scoring system with a prognostic effect for CSS for thymoma patients. The GPS was the only significant clinical scoring system in the univariable analysis of TCs.

Our study has some limitations due to its retrospective design and the single center character. In order to investigate a more homogeneous patient sample, only patients undergoing primary surgery for TETs with or without neoadjuvant treatment were included in this study. This selection bias does not allow the application of our findings to TET patients that were heavily pretreated or those with recurrent TETs.

Our results can only be applied in patients undergoing primary surgery for TETs. The scores still have to be evaluated for inoperable patients and those with recurrent disease.

The Masaoka-Koga staging system, the recently proposed TNM staging system, the WHO histological classification, the residual tumor classification or the extent of resection can only give reliable prognostic information according to clinico-pathologic and histologic features after surgical tumor resection. Further, they are purely tumor-related prognostic factors. The investigated parameters in this study are host-related (more likely a reaction of the host to the tumor) and give additional prognostic information to the essential tumor-related factors. The use of easily obtainable inflammation-based scores as host-related prognostic factors harbors great potential to narrow the gap that the application of tumor-related factors leaves in the outcome prediction of patients with TETs. The applicability of our findings warrants testing in inoperable TET patients and those that were heavily pre-treated or recurrent TETs.

The strengths of this study are the large number of patients with this disease, and the identification of the prognostic power of clinical inflammatory prognostic scores and their components.

The most promising predictive clinical scores of this single center retrospective experience warrant prospective multi-center study. Future studies under the patronage of ITMIG and the ESTS Thymic Working Group that are able to recruit the largest number of patients with TETs will provide a stronger evidence base.

## Material and Methods

### Ethics statement

Ethical approval was obtained from the ethics committee of the Medical University of Vienna. All participating patients gave their written informed consent, and all experiments were performed in accordance with the approved ethical guidelines.

### Study population

This study was conducted at the Departments of Surgery as well as Anesthesiology, General Intensive Care and Pain Management of the Medical University of Vienna. Patients with infections, COPD exacerbation or acute cardiac insufficiency were excluded from this analysis. There had to be at least four weeks between the last chemotherapy cycle or surgery and the date of serum analysis in order to avoid serum protein alterations due to prior treatment. We analyzed 184 patients with TETs who underwent surgical tumor resection between September 1999 and June 2018.

### Laboratory measurements

All blood samples were collected one day before surgery and were analyzed at the Department of Laboratory Medicine. CRP serum concentrations were measured using the latex-enhanced immunoturbidimetric assay (Roche, Mannheim, Germany) according to the manufacturer’s instructions. Serum albumin concentration was assayed from venous blood by the bromocresol green method with an accredited routine process. Complete blood cell (CBC) counts were measured on a Sysmex XE-5000 hematology analyzer (Sysmex Corporation, Kobe, Japan) within one hour of blood collection.

### Scoring systems

The calculations of the scoring systems were listed in Table 6. During clinical routine, anesthesiologists allocated patients into ASA 1–6 in order to estimate operative risk. In our cohort patients were only allocated to ASA 1–4. We classified patients with ASA 1 and 2 into ASA low and patients with ASA 3 and 4 into ASA high subgroups in order to dichotomize patients for survival and prognostic analysis.

**Table 6:** Clinical scoring systems.

Score	Prognosis	Definition
**ASA**^**a**^		
1		Patient is completely healthy and fit.
2		Patient has mild systemic disease.
3		Patient has severe systemic disease that is not incapacitating.
4		Patient has incapacitating disease with constant threat to life.
5		A moribund patient who is not expected to live 24 hours with or w/o surgery.
**ALI**^b^		BMIxALBUMIN/NLR
high	good	>26.1
low	poor	<26.1
**CFS**^**c**^		
0	good	CRP < 0.3 mg/dL and Fibrinogen < 452 mg/dL
1	poor	CRP > 0.3 mg/dL and Fibrinogen > 452 mg/dL
**GPS**^d^		
0	good	CRP < 1 mg/dL and Albumin > 35 g/L
1	intermediate	CRP < 1 mg/dL and Albumin < 35 g/L or CRP > 1 mg/dL and Albumin >35g/L
2	poor	CRP > 1 mg/dL and Albumin < 35 g/L
**HS-mGPS**^**e**^		
0		CRP < 0.3 mg/dL
1		CRP > 0.3 mg/dL and Albumin > 35 g/L
2		CRP > 0.3 mg/dL and Albumin < 35 g/L
**TET-aGPS**^**f**^		
0		CRP < 0.3 mg/dL and Albumin > 44 g/L
1		CRP > 0.3 mg/dL and Albumin < 44 g/L
**SII**^**g**^		neutrophil × platelet/lymphocyte [counts]
low	good	<655
high	poor	>655

A brief description of the ASA classification system and the components of composite scores are detailed together with the employed cut-offs.Cut-offs: The Youden Index was employed to define the optimal ALI cutoff values of 26.1 and 655 for the SII; the median pretreatment CRP (0.3 mg/dL) and the Youden Index of fibrinogen (452 mg/dl) was used to dichotomize patients into high and low CRP groups for the CFS score.

*ASA* American Society of Anesthesiology classification of Physical Health, *ALI* advanced lung cancer inflammation index, *NLR* Neutrophil-to-Lymphocyte Ratio*, GPS* Glasgow Prognostic Score, *HS-mGPS* the high-sensitivity modified Glasgow Prognostic Score, *TET-aGPS* thymic epithelial tumor adapted Glasgow Prognostic Score, *SII* systemic immune-inflammation index.

^a^Adopted from^43^.

^b^Adopted from^7^.

^c^Adopted from^17^ and^18^.

^d^Adopted^44^.

^e^Adopted from^12^.

^f^From^12,44^.

^g^Adopted from^10^.

The ALI score comprises BMI, serum albumin level and NLR. Patients’ height and weight were obtained from the medical records at admission one day before surgery. Absolute neutrophil count, absolute lymphocyte count and serum albumin concentration were obtained from the blood test analysis. The Youden-Index was used to determine the optimal cut-off value of 26.1 for predicting CSS.

In our previously published studies CRP and fibrinogen were analyzed as suitable markers to predict clinical outcome. Therefore, we summarized both acute phase proteins to develop a new suitable scoring system, the CFS especially for TETs. The mean pretreatment serum concentrations of CRP (0.3 mg/dL) and the previously published cut-off value for fibrinogen (452 mg/dL) were used to dichotomize patients into high and low CRP and fibrinogen groups, respectively^17^. Patients with elevated fibrinogen and CRP were categorized with CFS 1, while patients with only one or no abnormal value were allocated to CFS 0 cohort.

The GPS, the HS-mGPS and the TET-aGPS were composed of CRP reflecting the inflammatory and serum albumin levels reflecting the nutritional status.

Patients with a serum concentration of CRP (>1.0 mg/dL) and hypoalbuminemia (<35 g/L) were allocated to GPS 2. Patients with either CRP (>1.0 mg/dL) or hypoalbuminemia (<35 g/L) were allocated to GPS 1, and patients who had neither were allocated a GPS of 0 as previously published^9^. In order to perform survival analysis patients allocated to GPS 0 and 1 were summarized to GPS 0 1 and were compared with GPS 2. The HS-mGPS was calculated based on cut-off values of CRP (>0.3 mg/dL) and albumin (<35 g/l). The TET-aGPS was calculating using the mean CRP (>0.3 mg/dL) of our cohort and the Youden Index of albumin (<44.5 g/L) as cut-off values.

The SII was calculated from preoperative counts of peripheral blood platelets (P), neutrophils (N) and lymphocytes (L) per liter according to the equation: SII = P × N/L.

The optimal SII cut-off value of 655 was determined using the Youden-Index.

### Survival analysis

OS, CSS and FFR were used as the main study endpoints. OS was calculated from date of surgery to date of death of any cause. CSS was defined as death from TET, while FFR was calculated in patients after complete surgical resection (R0) from date of surgery to date of recurrence.

### Univariable and multivariable Cox regression

Univariable and multivariable Cox regression analyses were performed to evaluate the prognostic impact of clinical characteristics, including sex (male), age (<56 vs. >56 yrs), histology (TC vs. Thymoma), resection status (R0 vs. R1–2), myasthenia gravis (No), tumor stage (I–II vs. III– IV), ASA (I + II vs. III–IV), ALI (low vs. high), CFS (0 vs. 1), GPS (0 + 1 vs. 2), HS-mGPS (0 vs. 1 + 2), TET-aGPS (0 vs.1) and SII (low vs. high). In order to perform univariable cox regression analyses of the components of the scoring systems the median was used to dichotomize patients into low and high groups for albumin, BMI, NLR, CRP, fibrinogen, neutrophils, platelets and lymphocytes. For the components of the GPS, HS-mGPS and TET-aGPS the published and created cut-offs of CRP (>1.0 mg/dL), (>0.3 mg/dL) and albumin (<35 g/L), (<44.5 g/L) were used^9^.

### Scoring systems as Prognostic marker

The sensitivity, specificity, the PPV and NPV and binary logistic regression analysis were calculated for all scoring systems and single components.

### Statistical analysis

Parametric data are presented as mean ± standard deviation within result section. Survival analysis was performed using the Kaplan-Meier method and the log-rank test. To determine the optimal cut-off values, we calculated the Youden Index for ALI and SII score. All tests were two-sided and p-values below 0.05 were considered as statistically significant.

All statistical analyses were performed using SPSS (version 25.0; IBM SPSS Inc., IL, USA) and GraphPad Prism (version 5.0; GraphPad Sotfware Inc., California, USA).

## Supplementary information

Supplementary Figure

## Data Availability

All data generated or analyzed during this study are included in this published article (and its Supplementary Information files).
