# Supplementary material for: Clinical prognostic scores for patients with thymic epithelial tumors
Source: Sci Rep. 2019 Dec 9;9:18581. doi: 10.1038/s41598-019-54906-4 (PMC6901461; doi:10.1038/s41598-019-54906-4)
Supplement: Supplementary file 1 — Supplementary Figure [file 41598_2019_54906_MOESM1_ESM.pdf]

Clinical prognostic scores for patients with thymic epithelial tumors

Cecilia Veraar[1], Stefan Janik[2], Jürgen Thanner[3], Clarence Veraar[3], Mohammed Mouhieddine[1], Ana-Iris Schiefer[4], Leonhard Müllauer[4], Martin Dworschak[1], Walter Klepetko[3], Hendrik Jan Ankersmit[3,5] and Bernhard Moser[3]

[1]Department of Anesthesiology, General Intensive Care and Pain Medicine, Division of Cardiothoracic and Vascular Anesthesia and Intensive Care Medicine, Medical University of Vienna, Vienna, Austria

[2]Department of Otorhinolaryngology, Head and Neck Surgery, Medical University of Vienna, Vienna, Austria

[3]Division of Thoracic Surgery, Department of Surgery, Medical University of Vienna, Vienna, Austria

[4]Clinical Institute of Pathology, Medical University of Vienna, Vienna, Austria

[5]Head FFG Project "APOSEC", FOLAB Surgery, Medical University of Vienna, Vienna, Austria

Corresponding Author

Bernhard Moser MD PD Assoc. Prof. MBA FEBTS
Division of Thoracic Surgery, Department of Surgery, Medical University of Vienna. Waehringer Guertel 18-20. 1090 Vienna, Austria.
E-mail: bernhard.moser@meduniwien.ac.at; Phone +43 1 40400 67770

Conflict of Interest: The authors have no funding, financial relationships, or conflicts of interest to disclose.

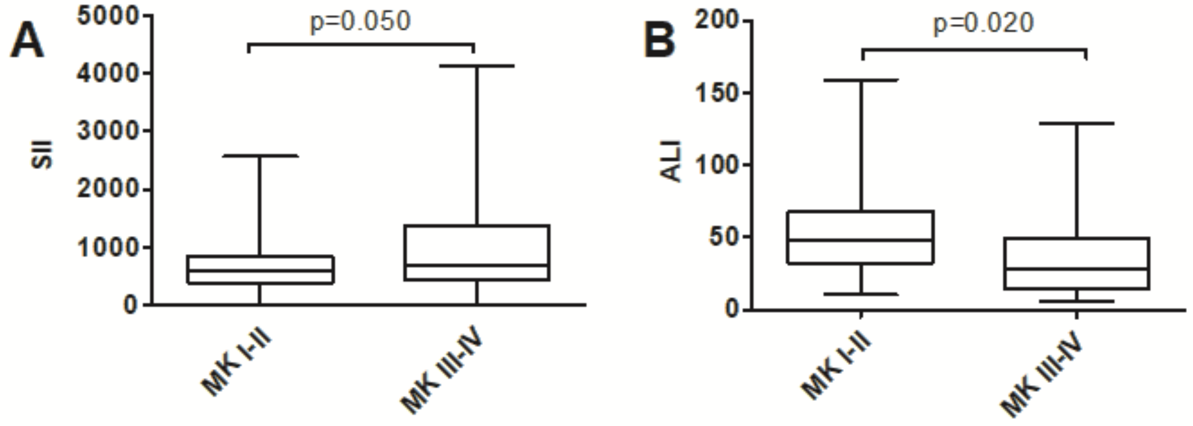

Supplementary Figure: Box plots of SII and ALI for patients with lower and higher tumor stages

Patients with *MK* III-IV had significantly higher SII (A) and lower ALI score (B) compared to those patients with MK I-II.

MK Masaoka-Koga tumor stage, *ALI* advanced lung cancer inflammation index, *SII* systemic immune-inflammation index.
